# Wearable Sensors for the Detection of Biomarkers for Wound Infection

**DOI:** 10.3390/bios12010001

**Published:** 2021-12-21

**Authors:** Alexandra Pusta, Mihaela Tertiș, Cecilia Cristea, Simona Mirel

**Affiliations:** 1 Department of Analytical Chemistry, Iuliu Hațieganu University of Medicine and Pharmacy, 4 Louis Pasteur Street, 400349 Cluj-Napoca, Romania; alexandrapusta@gmail.com (A.P.); mihaela.tertis@umfcluj.ro (M.T.); 2 Department of Medical Devices, Iuliu Hațieganu University of Medicine and Pharmacy, 4 Louis Pasteur Street, 400349 Cluj-Napoca, Romania; smirel@umfcluj.ro

**Keywords:** wearable biosensors, wearable sensors, wound infection, biomarker, electrochemical, colorimetric

## Abstract

Infection represents a major complication that can affect wound healing in any type of wound, especially in chronic ones. There are currently certain limitations to the methods that are used for establishing a clinical diagnosis of wound infection. Thus, new, rapid and easy-to-use strategies for wound infection diagnosis need to be developed. To this aim, wearable sensors for infection diagnosis have been recently developed. These sensors are incorporated into the wound dressings that are used to treat and protect the wound, and are able to detect certain biomarkers that can be correlated with the presence of wound infection. Among these biomarkers, the most commonly used ones are pH and uric acid, but a plethora of others (lactic acid, oxygenation, inflammatory mediators, bacteria metabolites or bacteria) have also been detected using wearable sensors. In this work, an overview of the main types of wearable sensors for wound infection detection will be provided. These sensors will be divided into electrochemical, colorimetric and fluorimetric sensors and the examples will be presented and discussed comparatively.

## 1. Introduction

Infection represents a major complication of both acute and chronic wounds, with a negative impact on wound healing, patient quality of life and economic resources [1,2]. Although the overall impact of wound infection is difficult to assess, it is estimated that surgical site infections in the UK alone affect 3–4% of surgery patients, cost an average €5800 per patient and cause an average mortality rate of 5% [3]. A retrospective study from 2018 in the USA indicated that around 8.2 million people suffered from infected or non-infected wounds. The highest costs of treatment were associated with surgical wounds and chronic foot ulcers. Due to factors such as an aging population and the rising incidence of diabetes and obesity, chronic wounds represent an increasingly problematic aspect of wound management [2]. In this context, it is vital to diagnose wound infection as early as possible in order to ensure the best treatment course for the patient. Currently used diagnostic methods are represented by clinical inspection and microbiological assays [4,5,6]. Despite being routinely used, these methods present several disadvantages such as inaccuracy, need for traumatic bandage removal and reliance on the physician’s expertise in the case of clinical examination. A few limitations of microbiological assays are long analysis times, invasive techniques in the case of microbiological assays carried out on biopsy tissue and failure to identify bacteria invading deep tissues in the case of swab cultures [4].

An alternative infection diagnosis method is the detection of certain biomarkers in the wound environment. In order to increase patient comfort and eliminate the possibly traumatic bandage removal process for clinical inspection, an ideal way of biomarker monitoring is the inclusion of wearable sensors for infection biomarkers in wound dressings. Numerous proof-of-concept examples of such sensors have been recently published, although, to date, none of these approaches has been clinically implemented on a large scale due to certain limitations. The development of wearable devices is faced with numerous challenges in respect to the materials used, energy sources and data transmission [5]. The incorporated materials need to be biocompatible and tailored so that they can conform to the curvilinear surface of the skin [5,6]. Moreover, they need to be flexible and resistant in order to ensure the user’s free movement [7]. Energy sources such as batteries are difficult to miniaturize and incorporate into wearable devices [6], but are indispensable for their operation and proper functioning, so it is necessary to find a solution to solve this inconvenience. Numerous challenges are also posed by designing appropriate and safe ways of wireless communication between the sensor and devices such as laptops and smartphones [5,7]. Such technologies are currently represented by Bluetooth [5,8], Near-Field-Communication (NFC) [9,10,11] and radio-frequency identification (RFID) [9,11]. Despite all these challenges, the development of sensors for point-of-care (POC) applications is a promising direction for the field of analytical techniques. The advent of wearable commercial devices for biological parameters (heart rate, blood pressure, movement) foreshadows the vital role wearable devices will play in personalized medicine.

The aim of this review is to present the latest contributions to the field of wearable and disposable sensors for wound infection biomarkers in the last five years. Biomarker detection represents a promising alternative for the diagnosis of wound infection. According to the National Institute of Health (NIH), a biomarker is a ‘characteristic that is objectively measured and evaluated as an indicator of normal biological processes, pathogenic processes, or pharmacologic responses to a therapeutic intervention’ [12]. The most important wound infection-related biomarkers will be briefly presented and the main techniques employed for their detection using wearable sensors will be discussed, with a focus on electrochemical methods. Examples of wearable sensors and smart dressings with both sensors and medicine included will be provided, compared and critically discussed.

## 2. Wound Infection Biomarkers

Recently, a plethora of potential biomarkers for wound infection have been identified. These biomarkers can be sub-divided into different categories, such as physiochemical parameters, enzymes, metabolites, signaling molecules, bacterial metabolites and bacteria [13,14,15] (Table 1). In spite of the numerous molecules that can be employed as targets, wearable devices have mostly been developed for a limited number of these biomarkers, with pH and uric acid being the most commonly studied. Other approaches have also been developed for oxygen and lactate, nitric oxide, hydrogen peroxide, enzymes, cytokines, pyocyanin or bacteria.

Normal skin pH varies between 4.2 and 5.6 [17], while in the case of infected wounds it has been proven to increase to slightly alkaline values [18,19], due to bacteria metabolites that are released into the wound environment [19]. These pH changes promote biofilm formation and increase the activity of tissue-damaging enzymes, thus having a negative impact on wound healing. The use of pH measurements alone for wound infection monitoring is deemed insufficient [18], but its use in combination with other biomarkers could be useful for early infection detection.

Uric acid (UA) is a metabolite produced in high quantities at the wound site due to metabolic pathway alterations [20]. In chronic wounds, such as leg ulcers, the wound milieu is generally hypoxic, which leads to the depletion of ATP and an increase in the production of purine metabolites, such as UA [21].

Poor vascularization at the site of chronic wounds leads to hypoxic conditions in the wound microenvironment [22]. Thus, oxygen concentration becomes a potential biomarker for monitoring wound status. Apart from UA, lactic acid is another metabolite that can be produced in high quantities in hypoxic conditions. Preliminary studies have demonstrated a connection between elevated levels of lactic acid and infected diabetic foot ulcers, making it a suitable biomarker for wound progression and infection monitoring [23].

Nitric oxide is a signaling molecule that is produced as a defense mechanism against bacterial infection [24]. Low concentrations of this biomarker have been associated with delayed wound healing and biofilm dispersal [25]. Similar to nitric oxide, hydrogen peroxide also plays a crucial role in wound healing and infection prevention, within well-defined concentrations. However, at high concentrations, it might prevent tissue regeneration and delay wound healing [26].

Neutrophil-derived enzymes are promising for wound infection diagnosis, being among the first biomarkers that appear early in the infectious process [16]. However, examples of wearable sensors for enzymes are scarce.

Elevated levels of cytokines such as tumor necrosis factor α (TNF-α), interleukin-6 (IL-6), interleukin-8 (IL-8) and transforming growth factor-β1 (TGF-β1) have been found in patients suffering from non-healing leg ulcers as compared to healing ulcers [27,28].

Pyocyanin is a bacterial metabolite secreted by *Pseudomonas aeruginosa*, a bacterium commonly found in infected wounds. Being produced only by this species, pyocyanin is considered a specific biomarker for *Pseudomonas aeruginosa* contamination or infection [29,30]. Naturally, the detection of bacteria itself in the wound fluid can be used to monitor infection status.

## 3. Detection Methods for Wound Infection Biomarkers

In this review, wearable electrochemical, colorimetric and fluorimetric sensors for wound infection biomarkers will be presented, with a focus on electrochemical sensors. The main advantages and disadvantages of each technique are summarized in Table 2.

Electrochemical methods have received great interest in the field of wearable sensors due to their numerous advantages. The advent of sensor fabrication techniques such as screen-printing or inkjet printing [31] on flexible substrates has enabled the rapid progress of electrochemical wearable sensors. These methods ensure inexpensive, reproducible, low-waste and large-scale production [31] of electrochemical sensors. Moreover, electrochemical sensors offer high sensitivity, quick analysis times and can be used for continuous monitoring [32]. In spite of this, several disadvantages still remain, such as difficulties in the miniaturization of potentiostats and the need for external power sources such as batteries [5].

The electrochemical techniques employed in wound biomarker detection are represented by amperometric, voltammetric and impedimetric methods. Amperometric methods rely on the application of a constant potential to the working electrode and the measurement of the generated current that is proportional to the analyte concentration [33,34]. Amperometric methods have been mainly employed in wearable sensors for the detection of UA [9,35,36,37], but also for the detection of oxygen or lactate [38]. In the case of voltammetry, a variable potential is applied at the working electrode, the resulting current is recorded and plotted against the potential variation [39,40]. Depending on the way in which the potential varies, voltammetric methods can be divided into cyclic voltammetry (CV), differential pulse voltammetry (DPV), square wave voltammetry (SWV) and others [39,40]. Voltammetric techniques have been used in wearable sensors for the detection of pH [37], UA, pyocyanin [25,29], nitric oxide [25], cytokines and *Staphylococcus aureus* [27]. In electrochemical impedance spectroscopy (EIS), the current generated by a change in the frequency of an input signal is measured [39]. EIS was generally used for the measurement of pH [36,41] as a wound infection biomarker.

Colorimetric methods have also been developed for wearable sensors for the detection of wound biomarkers, especially for wound fluid pH [11,42,43]. These methods rely on the incorporation of pH sensitive dyes into microbeads [11,42] or fibers [43] that are later incorporated into wound dressing materials. The pH indicator dyes change color in the pH range of wound fluid, allowing for both naked-eye estimations and pH measurements with the help of specially designed optoelectronic probes [11] or smartphones [42,43]. In certain examples [42,43], a simple photo of the sensor is taken using a smartphone and a specially designed algorithm correlates the color of the pH dye with a pH value, thus eliminating the need for optoelectronic probes. This approach has undeniable advantages, considering the ubiquitous nature of smartphones in today’s society.

Fluorimetric methods are another type of technique used in the design of wearable sensors for wound infection biomarkers. For fluorimetric detection, a beam of ultraviolet light is used to produce electron excitation in the analyte, causing it to emit visible light, a phenomenon called fluorescence. At low concentrations of the analyte, the intensity of the emitted light is proportional to the concentration of the analyte and thus this method can be used for analyte quantification. Bacteria [44], O_2_ [45] and H_2_O_2_ [26] have been detected using fluorescence methods. Bacteria was detected by using a dye that became fluorescent in the presence of bacteria [44], while the detection of H_2_O_2_ [26] and O_2_ [45] was done by employing substrates whose fluorescence was quenched in the presence of the analyte. Combined colorimetric and fluorimetric methods were also reported for pH monitoring [46].

## 4. Sensors for Wound Infection Biomarkers

Electrochemical, colorimetric and fluorimetric wearable sensors for wound infection biomarker monitoring are presented comparatively in Table 3 and Table 4.

### 4.1. Electrochemical Sensors

Chemical sensors were defined by The International Union of Pure and Applied Chemistry (IUPAC) as devices that provide the transformation of chemical response such as the concentration of a specific sample component, into an analytically useful signal which can be observed or recorded and used to detect the presence of the analyte in unknown samples [47]. Two individual but interdependent functional units are contained in a typical chemical sensor: a receptor and a transducer [47]. The receptor consists of either biomimetic elements such as aptamers, nanozymes or molecularly imprinted polymers (MIPs) [48] or biocomponents such as enzymes and antibodies in which case we have a biosensor [49]. Regardless of its nature, the role of the receptor is to transform the analyte concentration into a chemical or physical signal with a well established sensitivity and to provide high selectivity towards the target molecule in the presence of potentially interfering compounds [47]. The second functional unit of a chemical sensor is the physio-chemical transducer. Depending on the type of transducer, sensors can be classified as optical, calorimetric, piezoelectric and electrochemical [50,51].

Electrochemical sensors have the advantage of sensitivity, an important feature of electroanalytical methods, that can be combined with the selectivity of the receptor. In the case of electrochemical biosensors, the biocomponent recognizes its complementary analyte resulting in a catalytic or binding event that ultimately produces an electrical signal that is proportional to the analyte concentration and that can be monitored by the transducer [52]. Numerous applications have been tested for electrochemical sensors and biosensors in biomedical [53,54,55], environmental [55,56,57], industrial [55], and agricultural [58] applications. The sensitivity of the electrochemical sensors and biosensors can be greatly improved by using different nanomaterials such as graphene [59], carbon nanotubes, MXenes and metal nanoparticles [60]. Since nanomaterials represent a key element in the development of electrochemical wearable sensors, a quick overview of the most important types of nanomaterials employed in their fabrication will be briefly presented. Numerous extensive reviews [61,62,63,64,65] exist on this topic, so only the essential aspects will be detailed herein. Graphene is a two-dimensional nanomaterial composed of sp^2^ bonded carbon atoms, which displays remarkable properties like high surface area and excellent electrical and thermal conductivity [66,67,68]. Due to these properties, graphene is a widely used nanomaterial in countless sensor applications [66,67], including in the development of wearable sensors for wound infection biomarker monitoring [27]. Single-walled carbon nanotubes (SWCNT) are another type of carbon-based nanomaterial employed for sensor applications. They are considered a one-dimensional form of carbon that is formed by ‘rolling’ graphene into a cylindrical structure [68,69]. They present good chemical stability, strength and electrochemical conductivity. SWCNT were employed for electrode modification for the detection of lactate in order to increase electrode surface area and thus to increase sensitivity [38]. MXenes are a novel class of two-dimensional conductive nanomaterials, comprised of carbides, nitrides or carbonitrides of early transition metals. They present several properties which make them attractive for the design of wearable sensors. They are highly flexible and combine the high electrical conductivity of transition metals with the hydrophilic properties of their outer layer [35,70].

The wide diversity of nanomaterials that can be employed in the fabrication of electrochemical sensors, as well as the inherent advantages of electrochemical sensors have qualified them for applications in numerous fields, including wound monitoring.

**Table 3:** Examples of electrochemical wearable and disposable sensors for wound infection biomarker monitoring.

Detection	Analyte	Method	Linear Range	LOD	Matrix	Wireless Data Transfer	Ref
AMP	UA	C-SPE/PB/uricase/Chi on wound dressing	100–800 μM	NS	PBS	RFID, NFC	[9]
AMP	UA	Embroided ink coated/uricase thread (on gauze)	0–800 μM	NS	Simulated wound fluid	-	[35]
POT	pH	C/PANI on Ecoflex substrate	4–10	-	Standard pH buffer solutions, emulated wounds	-	[71]
POT	pH	ITOE/PANI, can be attached to bandage + NFC probe	4–10	-	Emulated wound and emulate infected wound	NFC	[10]
POT	pH	C-SPE/PANI on PET film, attached to commercial transparent tape	4–10	-	Standard pH buffer solutions	Bluetooth	[8]
POT	pH	C electrode on commercial bandage	2–13	-	Acidic and alkaline solutions	Using 2.4 GHz ISM band	[72]
EIS	pH	Screen-printed CuO NR on IDE	5–8.5	-	Buffer solution, DMEM	-	[41]
SWV	pH	Riboflavin/LIG/polyimide	2–8	-	Buffer solution	-	[73]
AMP	UA	Screen-printed carbon/uricase on omniphobic paper	0.22–0.75 mM	0.2 mM	PBS	Using 2.4 GHz ISM band	[36]
EIS	pH		5.5–8.5	-	Standard pH buffer solutions		
DPV	pH	LGG/MXene/PANI	4–9	-	Artificial wound exudate	-	[37]
AMP	UA	LGG/MXene/uricase	50–1200 μM	50 μM	Artificial wound exudate	-	
SWV	UA	CNT/PA	100–1000 μM	NS	Simulated wound fluid	-	[29]
	Pyo		0.10–100 μM	0.1 μM	Simulated wound fluid, bacteria culture media		
SWV	UA	CUA	100–700 μM	1 ± 0.4 μM	PBS, simulated wound fluid	-	[25]
	Pyo		1–250 μM	1 ± 0.5 μM	PBS, Simulated wound fluid, bacteria culture		
	Nitric oxide		1–100 μM	0.2 μM	PBS, simulated wound fluid, eukaryotic cell culture		
AMP	Oxygen	AuE/Nafion/PDMS on wound dressing	58.5–178 [O_2_]%	NS	PBS	-	[38]
AMP	Lactate	AuE/PB/SWCNT/Chi/LO/SWCNT/Chi on wound dressing	0.1–0.5 mM		PBS	-	
SWV	TNF-α	AuE/AuNPs-GP/Apt-MB	0–2 ng/mL	NS	Spiked serum, mice wounds, wound exudate	Bluetooth	[27]
	IL-6		0–30 ng/mL				
	IL-8		0–30 ng/mL				
	TGF-β1		0–150 pg/mL				
	*Staph. aureus*		0–1 × 10^9^ CFU				
	pH	PANI/AuE	4–9				

AMP—amperometry; POT—potentiometric; UA—uric acid; C-SPE—carbon screen-printed electrodes; PB—prussian blue; Chi—chitosan; NS—not specified; PBS—phosphate buffer saline; RFID—radio frequency identification; NFC—Near-Field Communication; PANi-EB—polyaniline emeraldine base; PANI—polyaniline; ITOE—indium tin oxide electrode; PET—polyethyleneterephtalate; EIS—electrochemical impedance spectroscopy; NR—nanorods; IDE—interdigitated electrodes; DMEM—Dulbecco’s Modified Eagle Medium; SWV—squarewave voltammetry; LIG—laser-induced graphene; DPV—differential pulse voltammetry; LGG—laser guided graphene; Pyo—pyocyanin; CNT—carbon nanotube; PA—polyacrylamide; CUA—carbon ultramicroelectrode arrays; AuE—gold electrode; PDMS—polydimethylsiloxane; SWCNT—single-walled carbon nanotubes; LO—lactate oxidase; TNF-α—tumor necrosis factor α; IL-6—interleukin 6; IL-8—interleukin 8; TGF-β1—transforming growth factor-β1; AuNPs-GP—gold nanoparticles graphene nanocomposite; Apt—aptamer; MB—methylene blue; CFU—colony forming units.

**Table 4:** Examples of colorimetric and fluorimetric wearable and disposable sensors for wound infection biomarker monitoring.

Detection	Analyte	Method	Linear Range	LOD	Matrix	Wireless Data Transfer	Ref
COL	pH	pH indicator dye embedded in hydrogel wound dressing + optoelectronic probe	6.4–8.4	-	Standard pH buffer solutions	RFID, NFC	[11]
COL	pH	pH indicator dye embedded in alginate microfibers	6.2–8.2	-	Agarose and standard pH solutions on pig skin	Smartphone photo	[42]
COL	pH	Curcumin embedded in PCL fibers	6–9	-	Standard pH buffer solutions	Smartphone photo	[43]
COL + FL	pH	O-QDs on commercial medical cotton cloth	5–9	-	PBS	-	[46]
COL	Bacteria	MB embedded in PVA/CMC	NS	NS	Bacteria suspension	-	[74]
FL	Bacteria	Florescent dye vesicles embedded in GelMA	NS	NS	Mice wounds	-	[44]
FL	H_2_O_2_	Eu CP/PAN fiber mats	20–200 μM	NS	Standard solutions, animal wounds	-	[26]
FL	O_2_	Ru(dpp)_3_Cl_2_/paper	5–26 ppm	NS	Oxygenated water, mice wounds	-	[45]

COL—colorimetric; RFID—radiofrequency identification; NFC—Near-Field Communication; PCL—polycaprolactone; FL—fluorimetric; O-QD—orange-emitting quantum dots; MB—methylene blue; PVA—polyvinyl alcohol; CMC—carboxymethylcelulose; NS—not specified; GelMA—methacrylated gelatin; Eu CP—Europium coordination polymers; PAN—polyacrylonitrile; Ru(dpp)_3_Cl_2_—Tris(4,7-diphenyl-1,10-phenanthroline) ruthenium(II) dichloride.

#### 4.1.1. Electrochemical Uric Acid Sensors

UA represents the final circulating metabolite resulted from the purine metabolism. It is a relevant marker for cardiovascular and renal disease, and has been adopted as a detection indicator for gout. Also, UA has been found in high concentrations in chronic wounds, being correlated with the evolution of the wound.

For the electrochemical detection of UA by using electrochemical sensors, the property of this compound to be easily oxidized in aqueous medium is exploited [75]. Thus, several electroanalytical detection strategies were optimized for UA by CV, DPV and amperometry, as fast, simple, and highly sensitive methods. Different sensing materials have been employed in order to maximize both the sensitivity and selectivity of UA detection such as graphene, graphene-composite, carbon nanotubes, metal oxides and metallic nanoparticles-based materials, most of which proved to be suitable for this purpose, primarily due to their properties, especially those related to the high surface to volume ratio, large electrochemically active surface and the high speed of electron transfer.

**Figure 1:**
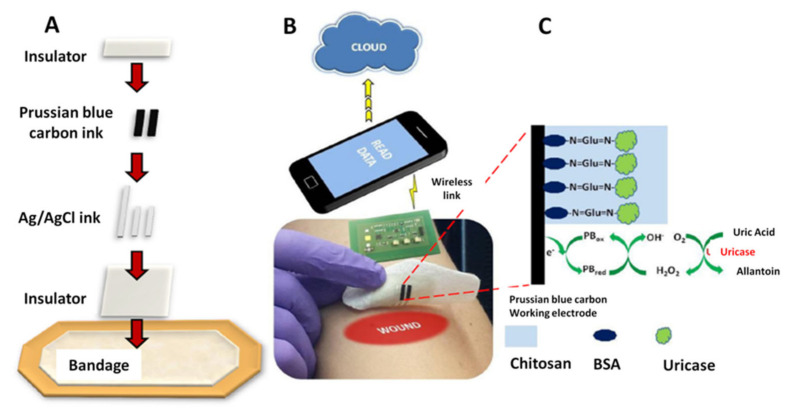
(**A**) Screen printing of the smart bandage. (**B**) Wearable potentiostat used for the operation of the sensor. The analytical signal used for UA detection and quantification is transferred to a computer or Smartphone via the wireless system. (**C**) Schematic representation of amperometric detection of UA with uricase immobilized on Prussian blue (PB) modified working electrode. BSA = bovine serum albumin. Reprinted with permission from Reference [9]. Copyright 2021 Elsevier.

A new type of smart bandage was designed for the determination of UA as an important biomarker for wound infection. The UA levels have been monitored directly on a wound dressing using an amperometric biosensor printed on a bandage. The elaboration steps and the operating principle of the electrochemical biosensor are presented in Figure 1 [9].

The amperometric detection of the target analyte at a low potential was catalyzed by uricase in the presence of Prussian blue (PB). The smart biosensor was screen-printed on a bandage and was operated by using a custom made wearable potentiostat which allowed wireless (RFID and NFC) data transfer to any type of smart devices, being very promising for healthcare applications since their use will generate lower costs and less stress and pain for the patients [9].

A similar approach using uricase-modified electrodes was described for UA detection. Here, however, the sensors were fabricated using an embroidering process. Conductive ink-coated polyester thread was embroidered on gauze and then covered in uricase solution to obtain the UA sensor. Compared to screen-printing, this approach leads to an increase in the mechanical resistance of the sensor. Similar to the previous approach, the detection of UA was done by amperometry. The method was also tested on simulated wound fluid, as opposed to the previous approach, where only spiked phosphate buffer samples were tested. A disadvantage of this method is represented by the lack of wireless communication and data transfer [35].

#### 4.1.2. Electrochemical pH Sensors

Determining the pH value of the wound environment is a topic of great interest to researchers; so many wearable sensors for this parameter have been developed so far. pH is among the key parameters for monitoring chronic wounds, since its switch from acidic to alkaline is typically interpreted as an indication of bacterial infection. This important property recommends this marker for prescreening of chronic wounds [46]. Monitoring of wound pH is critical for interpreting wound status, and early identification of wound infection or non-healing wounds is very useful for the administration of treatment at exactly the right time.

**Figure 2:**
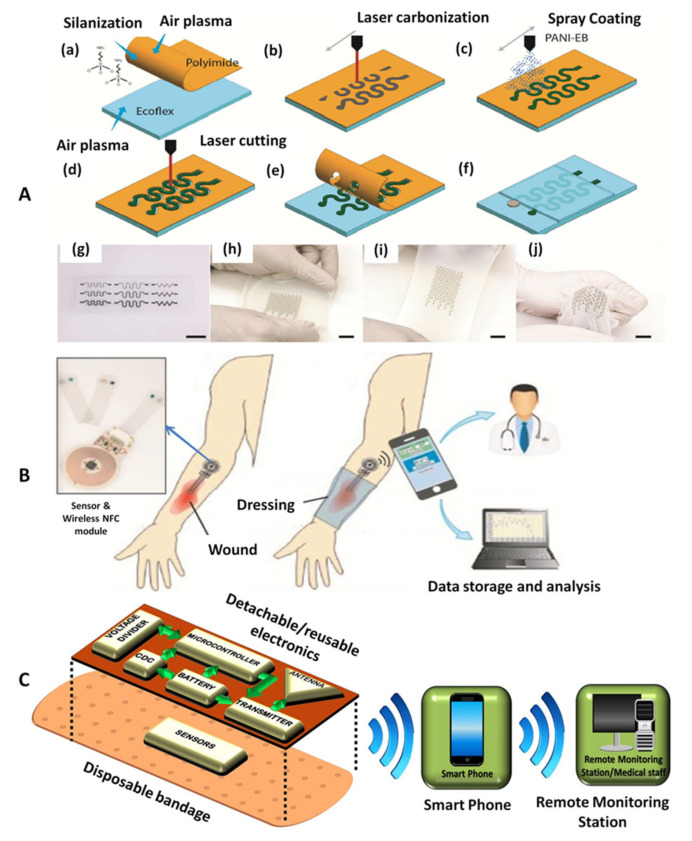
(**A**) Schematic illustrations of the fabrication process and images of the stretchable pH sensor with serpentine interconnects: (**a**) Polyimide (PI) layer fixed on Ecoflex substrate via silanization, (**b**) laser carbonization of carbon traces, (**c**) PANI coating of the carbon traces, (**d**) PI laser treatment, (**e**) excess PI removal, (**f**) Ecoflex insulation of the interconnects and Ag/AgCl and solid electrolyte deposition, (**g**) photograph of various stretchable PANI/C-PI interconnect designs, (**h**–**j**) images illustrating pH sensors being stretched. Reprinted (adapted) with permission from Reference [71] Copyright 2021 American Chemical Society. (**B**) Flexible wireless wound pH monitoring system using NFC communication. Left: The image presenting wireless NFC module. Reprinted (adapted) with permission from Reference [10]. Copyright 2021 Elsevier. (**C**) The schematic representation of a smart bandage assembly used for pH monitoring. Adapted from Reference [72].

A wearable electrochemical potentiometric sensor for pH with good perspective for POC applications was designed. This sensor consists of a pH sensitive working electrode and a liquid-junction-free reference electrode, having stretchable and conductive properties and of a pH sensitive membrane based on a conductive layer of polyaniline (PANI). The optimized sensor demonstrated good stability and biocompatibility. The protocol applied for the fabrication process is illustrated in Figure 2A. The process starts by generating a thin film of silanized polyimide (PI) on a plasma treated elastic support. Carbon-based porous conductive patterns were directly pyrolyzed onto the PI side of the film, and coated with PANI for increased mechanical resistance as well as for highly sensitivie pH determination [71].

Figure 2B shows an illustration of a wireless pH monitoring system suitable integration into standard wound dressings, which was successfully employed for pH measurements. In this approach, the pH monitoring device presents two major components: a disposable, flexible and transparent pH sensor and a reusable, flexible wireless NFC circuit. Compared to other approaches [36], this wireless tag does not require the use of batteries making it easy to wear and light-weight. The pH measurement is based on open circuit potential (OCP) tests between the Ag/AgCl reference electrode and a PANI-based pH-sensitive working electrode. The pH sensitivity of PANI is due to the protonation and de-protonation of nitrogen atoms in its polymer chain. In acidic solutions, PANI is protonated, which increases its OCP with respect to the reference electrode. In contrast, exposure to alkaline solutions will induce de-protonation and result in a decrease in the OCP. The sensor demonstrated acceptable mechanical resistance, lower to that of the previous approach, however both sensitivity and correlation coefficient exhibited higher values in this case. The sensor could be used for maximum one day after its fabrication due to some stability limitations [10].

Another low-cost detection system has been designed to detect bleeding, pH and external pressure on the wound, by using a combination of a disposable part and a reusable part attached to a disposable bandage (Figure 2C). All the electronics were integrated on a flexible medical tape which can be detached and reused while wireless communication is realized via an inkjet printed antenna. A smartphone can be further used to monitor wound progression regardless of the patient’s location, while healthcare providers will receive this information either via the mobile network or via the internet [72].

An impedimetric flexible sensor based on cupric oxide (CuO) was also reported for pH measurement. CuO is a transition metal oxide that acts as a p-type semiconductor [76]. It is stable, environmentally friendly [76] and enables electron transfer at low potential values [41]. Due to these advantages, CuO was used in the fabrication this sensor. Two configurations were used, CuO nanorods and nanoflowers and the nanorod-based electrodes demonstrated higher sensitivity compared to the nanoflower-based ones [41]. The sensors consisted in screen-printed interdigitated electrodes on flexible substrates. The sensors were tested in a narrower pH range (5–8.5) compared to other techniques, demonstrated certain stability problems and did not include a wireless transmission system. However, their flexible nature makes them promising for future studies regarding the development of portable and wearable systems [41].

pH sensors were also developed by using laser-induced graphene (LIG) obtained after a laser treatment of a PI flexible substrate. The detection was performed using optimized potentiometric and voltammetric procedures, the latter providing better results. Similar to the approach of Manjakkal [41] this method does not allow for wireless data transmission, but the substrate flexibility could be used for POC devices in the future. Tests were carried out only on buffer solutions, with no real samples being tested. The pH response was tested on a wide range of pH (2–8), however, the pH of infected wound exudate is alkaline, so further tests in more alkaline media should be performed in order to ensure the applicability of the method in wound monitoring. An additional disadvantage would be the need for riboflavin as a redox probe. The addition of this molecule could provide difficult in real-life measuring conditions [73].

#### 4.1.3. Electrochemical Sensors for Multiple Analytes

In most cases, biomarkers are not specific to a particular condition, but can be overexpressed or underexpressed in several different conditions. Thus, in order to be able to use the rapid detection of biomarkers from biological fluids for treatment purposes, it is preferable to detect two or more biomarkers and to establish a pattern of disease based on multiple detection. Studies on the detection of two or more markers or biomarkers in wounds have already been reported in the literature.

**Figure 3:**
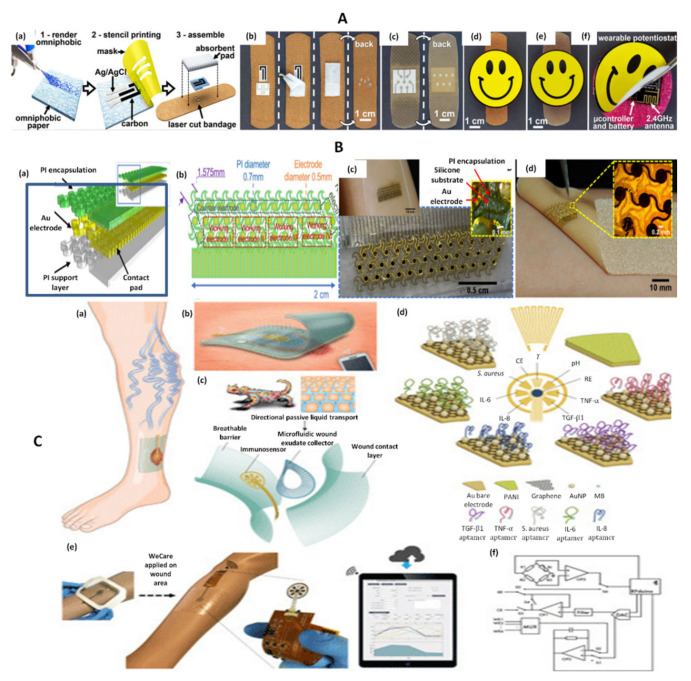
(**A**) Fabrication protocol for omniphobic paper smart bandage (OPSB) fabrication and assembly process. (**a**) Schematic diagram of the fabrication of OPSBs with: (1) Whatman#1 paper rendered omniphobic by spraying a 2% solution of RF SiCl_3_ in isopropyl alcohol; (2) stencil printing is used to pattern flexible conductive electrodes using carbon and Ag/AgCl inks; (3) openings are laser cut on the adhesive layer of the bandage to interface the wearable potentiostat with the paper-based sensors in the OPSB. OPSBs are assembled by placing the paper-based sensors between the adhesive layer and the absorbent pad of the commercial bandages. (**b**) OPSBs used to monitor UA and pH levels in open wounds. (**c**) OPSBs used for the early detection of pressure ulcers. (**d**,**e**) Interfacing of the wearable potentiostat with OSPBs for monitoring open wounds and detecting 12 pressure ulcers, respectively. (**f**) Packaging of the electronics in the rechargeable, wearable Potentiostat Reprinted (adapted) with permission from Reference [36] Copyright 2021 Elsevier. (**B**) Skin-inspired gold electrodes (**a**); schematic diagram of the flexible device (**b**); schematic diagram with dimensions (**c**); image of the sensor on polymeric wound dressing (Walgreens—Silicone Scar Sheets) (**d**); image of the sensor on a textile-silicon bandage (ScarAway—Silicone Scar Sheets) Reprinted (adapted) with permission from Reference [38]. Copyright 2021 Elsevier. (**C**) Schematic representation of a multiplexed immunosensing system for chronic wound monitoring. (**a**) Illustration of a biomarker analytical dressing applied onto an open wound of patients with venous ulcer for in situ surveillance. (**b**) Illustration of a thin, soft, biomarker analytical dressing (VeCare) that allows normal skin function allowing the penetration of oxygen inside but also the evacuation of moisture vapor outside. Data was wirelessly transmitted to a mobile system over Bluetooth Low Energy. (**c**) Envisioned biomarker analytical dressing constituting of a perforated wound contact layer, a microfluidic wound exudate collector, an immunosensor, and a breathable barrier. The microfluidic collector was inspired by the skin of Texas horned lizard enabling predetermined flow direction toward the lizard’s snout defying gravity. (**d**) Schematic representation of the sensors applied for the detection of TNF-α, IL-6, IL-8, TGF-β1, *S. aureus*, pH, and temperature. PANI = polyaniline; MB = methylene blue; RE = reference electrode; CE = counter electrode. (**e**) VeCare prototype for envisioned chronic wound monitoring. The prototype was applied to a leg dummy as a demonstration. The immunosensor interfacing with a wireless portable analyzer fabricated on a flexible printed circuit board. A mobile application providing a graphical user interfaceas a one-stop patient’s profiles, medical records, data recording, data analysis, and result visualization system is shown. (**f**) Hardware block diagram for the VeCare platform. WE1 = working electrode 1; MUX = multiplexer Adapted from Reference [27] (Open Access).

For example, a combined strategy for the simultaneous electrochemical detection of UA and wound pH was reported (Figure 3A). The selected electrodes were screen-printed onto omniphobic paper and incorporated into commercially available bandages. Similar to previously described methods, UA was detected using uricase-modified electrodes and an electrochemical strategy based on amperometry. For pH determinations, EIS was used. Two silver electrodes were printed and a layer of PANI was deposited between them. PANI changed its conductive properties in relation to pH, thus allowing pH measurements to be made. A portable, rechargeable and reusable potentiostat was designed and incorporated into the device. The potentiostat allowed wireless transmission of data to smart devices, thus being convenient for POC wound monitoring. To prove the practical applicability of the omniphobic paper-based sensing device, it was also successfully adapted for tissue impedance measurements for the early detection of pressure ulcers in mice [36].

A different strategy for simultaneous pH and UA monitoring was developed based on laser-guided graphene modified with titanium-based MXene nanosheets. Uricase was used for the amperometric detection of UA, while PANI was employed for the DPV monitoring of pH. Compared to the previous approach, this method achieved a lower limit of detection for UA and demonstrated a linear response on a wider pH range. In spite of these advantages, no wireless data transfer method was developed [37].

Another proof-of-concept dual electrochemical monitoring strategy was reported by Jarosova et al. [29]. In this approach, UA and pyocyanin were detected by SWV using inkjet printed carbon nanotube electrodes that were covered in a thin layer of polyacrylamide to reduce fouling. Inkjet printing has several advantages compared to screen-printing, such as higher precision and reproducibility. The obtained electrodes were disposable, but were not integrated into a completely wearable system, thus limiting the feasibility of this method for POC devices. However, the electrodes demonstrated good stability and tests on simulated wound fluid and bacterial cultures showed promising results [29].

Simultaneous detection of UA, pyocyanin and nitric oxide was also performed using flexible carbon ultramicroelectrode arrays (CUA). Similar to the previous approach, SWV was used for detection of the three biomarkers. CUAs were obtained by a complex process, involving electron-beam deposition of copper, lithography and atomic layer deposition on a flexible polyethyleneterephtalate (PET) substrate. Despite being flexible, the obtained electrodes were not integrated into a completely wireless device and the potentiostat used was not a portable one. Extensive analyses were carried out in PBS, simulated wound fluid and bacterial cultures (for pyocyanin) and certain differences were observed in the electrochemical behavior of the analytes in phosphate buffer saline (PBS) compared to simulated wound fluid. Moreover, some differences between the results of this study compared to those reported by Jarosova et al. became apparent [25].

Another example of dual-analyte monitoring sensor was reported for lactate and oxygen (Figure 3B). In this case, a flexible, skin-like mesh structure was developed as follows: chrome and gold were deposited onto a silicon wafer by photolithography, followed by PI spin coating. The sensor was printed on water-soluble tape and finally transferred onto silicon commercial bandage. For lactate detection, the gold electrode was modified with SWCNT to increase surface area, PB to improve sensitivity, lactate oxidase to increase selectivity towards lactate and chitosan for enzyme immobilization. For oxygen detection, the gold electrode was covered in Nafion and polydimethylsiloxane, which acted as an oxygen selective membrane. The sensor displayed skin-like mechanical properties, however no wireless data transfer device was used in this approach [38].

A suggestive example to illustrate multiple biomarker detection from wounds refers to the VeCare device which was designed and developed to allow the detection of no less than six relevant markers or biomarkers for the monitoring of venous ulcers (Figure 3C). The selected markers were IL-6, IL-8, TNF-α, TGF-β1, pH and *Staphylococcus aureus*, VeCare being thus a promising prototype sensor for the detection of a multitude of analytes. This complex approach consisted of a microfluidic cell that was designed to collect wound exudate on the surface of an array of sensors. The cytokine and bacteria sensors consisted of gold electrodes modified with AuNPs, graphene and the corresponding aptamer for each target. The gold nanoparticles (AuNP)—graphene composite combined the high conductivity of graphene with the high current density and quick mass transport offered by AuNPs in order to improve the sensitivity and overall functionality of the aptasensors [27]. The aptamers were modified at one end with thiol groups to ensure covalent bonding to the gold surface and at the other end with methylene blue (MB), which acted as a redox probe. Once the analyte was captured, the distance between the MB probe and the electrode increased, thus leading to a decrease in the SWV signal. The pH sensor consisted of a gold electrode modified with PANI. A wireless electrochemical analyzer was incorporated and Bluetooth transmission was employed to send the obtained data to a smartphone equipped with a specially designed application. The method was extensively tested both in vitro and in vivo, on mice wounds. Apart from all these advantages, the highlight of this approach was the clinical testing of the sensors on wound exudates from five patients. The device was not worn by the patients, but, instead exudate was collected with a syringe and placed on the surface of the sensors. Despite this, this approach is a valuable step forward in the development and clinical application of wearable sensors [27].

### 4.2. Colorimetric Sensors

Colorimetric sensors are important representatives of optical sensors that change their color when influenced by external stimuli. The trigger for this change can be any variation in a physical or chemical parameter found in the system. Colorimetric sensors represent a widely used category of sensors and find multiple applications in areas such as biomedicine, environmental field, food analysis and quality control, security, etc. This is mainly primarily due to their simple and easy operation, fast response, high sensitivity and selectivity. Moreover, the most important aspect related to colorimetric sensors is that the analytical signal based on which the detection and even quantification of the target analyte occurs, can be observed with the naked eye. The recent progress of colorimetric sensing strategies involves the use of nanomaterials and biomimetic elements, such as metal oxide/sulfides-based, metal-based, carbon-based, and aptamer-conjugated magnetic nanomaterials—the so-called nanozymes, which present enzyme-like catalytic activity [77,78,79].

#### Colorimetric pH Sensors

An optical pH detection approach consisted in the embedding of a pH-sensitive dye in a commercial hydrogel wound dressing. A specially designed optoelectronic probe was used to determine the pH and to wirelessly transfer the data via RFID or NFC to a computer or smartphone. The probe consisted of a small, portable device that incorporated a LED and photodiode, as well as a memory of 1 MB that can store data from up to 32,000 pH measurements. The performance of the smart bandage was compared to that of a standard pH-meter and no statistically relevant differences were observed. The device was only tested on standard pH buffer solutions, with no tests on biological fluids [11].

**Figure 4:**
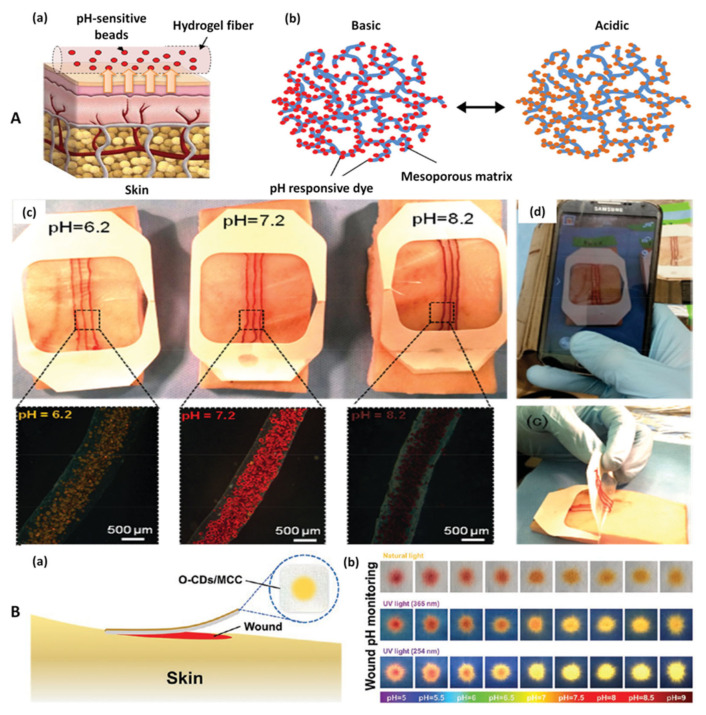
(**A**) Fabrication of pH-sensing microfibers. A schematic illustration of the pH-sensing hydrogel microfibers designed for long-term epidermal monitoring (**a**) and the action mechanism of the mesoporous polyester particles containing pH-responsive dye with electrostatic interaction to the solid matrix of the mesoporous particles (**b**). Fabricated wound dressings are placed on pieces of pig skin sprayed with solutions of different pH. The images confirm sufficient visual difference for identifying the variation in the skin pH for the range relevant to the values in chronic wounds. The insets are showing the images of the engineered fibers in the corresponding solutions (**c**). Pictures taken using a smartphone for determining the pH of the substrate (**d**). Reprinted (adapted) with permission from Reference [42]. Copyright 2021 John Wiley and Sons. (**B**) Schematic and conceptual view of the orange-emissive carbon quantum dots immobilized on common medical cotton cloth (O-CDs/MCC) in practical application (**a**). Photographs showing color appearance under natural light and fluorescence images under UV light (excited at 365 and 254 nm) of the O-CD/MCC treated by buffer solution at different pH value (**b**). Reprinted (adapted) with permission from Reference [46]. Copyright 2021 John Wiley and Sons.

A similar method using a pH sensitive dye (brilliant yellow) was developed by Tamayol et al. (Figure 4A). In this case, the dye was incorporated into mesoporous particles that were embedded into alginate microfibers. The modified fibers were then attached to medical tape to create a wearable sensor that was tested on pig skin grafts impregnated with agarose and solutions of different pH values. Compared to the previous method, this approach has the advantage of using a smartphone for data acquisition. A photo of the wearable sensor can be taken using a smartphone and the color of the dye is analyzed using a specially designed algorithm that correlates the color with the pH value. This eliminates the need for special devices for data analysis and makes the approach feasible for POC use. These pH sensitive fibers developed in this study can be added to any commercial wound dressing to provide real-time information about the wound condition without the need for expensive instrumentation [42].

A similar approach relied on the pH sensitive properties of curcumin [43]. Curcumin is a natural colorant which is extracted from turmeric (*Curcuma longa*) and exhibits the property to change color from yellow to brown-orange with the change of pH from acidic to alkaline media [80]. Curcumin was embedded into electrospun polycaprolactone (PCL) fibers that could be tailored into a variety of shapes and sizes. The color change could be monitored by the naked eye, as well as with the help of a smartphone. A photo of the fibers was taken, the colors were analyzed and a linear correlation between pH and the green component of the color was found, thus enabling the correlation between pH and color.

A combined fluorimetric and colorimetric pH detection strategy was also reported. Orange emissive carbon quantum dots (O-CDs) were synthesized using an assisted heating method. Quantum dots (CDs) are zero-dimensional nanomaterials that possess a wide range of properties such as high UV light absorption, fluorescence, low toxicity and biocompatibility which make them attractive for sensor applications in a wide variety of analytical fields [81,82]. The synthesized O-CDs were embedded into commercial cotton cloth and the O-CDs-modified cloth exhibited pH-dependent changes in both fluorescence and color in a wide pH range (5–9) (Figure 4B). Compared to previously described approaches, short-term biocompatibility studies were performed and good results were obtained. Moreover, lack of interference from blood components, high stability and similar degree of comfort compared to unmodified medical cotton cloth were demonstrated. This approach does not include a wireless data acquisition system and relies on the use of UV lamps which might not be readily available for patients. However, the complex analyses that were carried out demonstrated its promising characteristics [46].

### 4.3. Fluorimetric Sensors

Fluorimetric sensors are based on the principle of the corresponding method, namely fluorescence spectroscopy (fluorimetry), a method in which the fluorescence of the sample is analyzed.

Fluorimetry is considered to be one of the most reliable analytical techniques, mainly due to its simplicity, sensitivity, and low cost, thus being very commonly employed in the majority of quality control laboratories [83].

A fluorimetric method was developed for the semi-quantitative detection of H_2_O_2_ using Europium-based coordination polymers (EuCPs) that were attached to electrospun polyacrylonitrile (PAN) fiber mats that acted as a smart bandage. The EuCPs were synthesized using a hydrothermal method. An interesting property of EuCPs was their ability to induce neoangiogenesis and thus promote wound healing in mice [26]. The fluorescence of the EuCps/PAN mats was quenched upon H_2_O_2_ addition and a visual estimation of H_2_O_2_ concentration could be performed. The variation of fluorescence with concentration was linear in the domain 20–200 μM, however, the coefficient of determination (R^2^) had a relatively low value of 0.927. Moreover, the method required the use of a fluorescence spectrophotometer, which limits its applicability as a POC device.

A smart dressing that could be used for both delivery and detection of oxygen in hypoxic wounds was developed [45]. For oxygen detection, a ruthenium-based dye was inkjet printed onto a paper substrate. The dye could be excited with visible light to emit fluorescence, as opposed to other approaches which required UV light excitation [44]. This represents an advantage, since UV lamps might not be available for the general population. In the presence of oxygen, the fluorescence of the ruthenium dye was quenched and the fluorescence decay rate could be correlated with oxygen concentration. The detection was done using an external optical probe. Mechanical characterization, cytotoxicity assays and in vivo biocompatibility and functional assays were carried out and all demonstrated promising results.

### 4.4. Theranostic Approaches

Smart-dressing devices, most often represented by smart-badges, or smart-patches, can have double applicability. In this case, in addition to the built-in sensors for the detection of markers or biomarkers relevant to the wound healing process, they also contain drugs for wounds treatment. Medicines incorporated in the dressing are released immediately or for a longer time, which can be beneficial for the healing process. Thus, with a single device it is possible to ensure both the treatment of wounds and the monitoring of the evolution of the wound during treatment.

**Figure 5:**
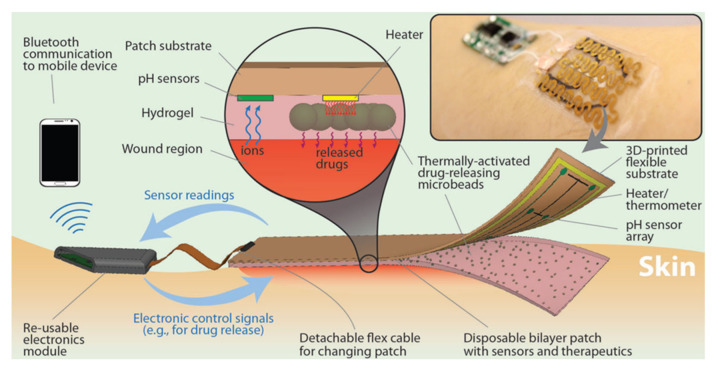
Schematic and conceptual view of the automated smart bandage. The bandage was comprised of an array of flexible pH sensors and a flexible heater to trigger thermo-responsive drug carriers containing antibiotics. Thermo-responsive drug carriers were embedded in a layer of alginate hydrogel which was cast around the pH sensors and on top of the flexible heater. The sensors and heater were connected to an electronic module that could record the sensors signal and power the heater if needed. The electronic module could also communicate wirelessly to computers and smartphones Reprinted (adapted) with permission from Reference [8]. Copyright 2021 John Wiley and Sons.

A smart bandage was designed, with several subunits such as pH and temperature sensors [8]. The device was equipped with thermo-responsive drug carriers based on poly(N-isopropylacrylamide) embedded in a hydrogel patch, and with a temperature sensor, which could detect wound inflammation. This patch was fixed onto a flexible heater operated via integrated electronics and wireless transmission was assured to read the data from the sensors (Figure 5). The entire construct was attached to commercial transparent medical tape to form a wearable platform that was very thin and flexible. The platform was built in a way that the ensured that the sensing modules and the integrated heater were low-cost and disposable, while the electronics were reusable.

An innovative theranostic wound dressing comprised of two layers was developed by Zhou et al. [44]. The first layer consisted of lipid vesicles containing antimicrobial agents, while the vesicles in the second layer contained a fluorescent dye in a concentration high enough to quench fluorescence. The second layer was designed for pathogenic bacteria sensing. In the presence of pathogenic bacteria, the lipid layers were disrupted by bacterial toxins and enzymes, releasing the dye and diluting it, thus activating fluorescence. Similarly, the vesicles in the first layer were also disrupted, allowing the release of antimicrobial agents into the wound. A disadvantage of the method is represented by the need for a UV light source for fluorescence detection. However, the study provides important and complex information and the developed dressing was also tested in vivo on mice wounds. Despite of the platform’s high stability, its use in vivo is time-limited by the complex wound environment, which quenches fluorescence and does not allow for pH monitoring throughout the whole healing process.

## 5. Perspectives and Conclusions

In the last years, technology and smart devices have rapidly become an integrated part of our everyday lives. Sensors for biological parameters such as gait, pulse or blood pressure have already been incorporated into smartphones and commercial wearable devices. In this context, it is natural to expect that wearable and smart sensors will start playing a crucial role in the future of personalized medicine. The field of wound infection diagnosis and monitoring makes no exception to this general trend. By measuring the changes in different biomarkers present in the wound milieu, wearable sensors for wound infection represent the next generation of devices that could be used in the future for a more rapid and accurate diagnosis of infected wounds.

The development of wearable sensors for wound infection biomarker monitoring can provide numerous advantages both for the patient and for healthcare personnel. Constant monitoring of wound environment changes can increase patient comfort and compliance by reducing the need for traumatic bandage removal for wound inspection. By reducing disruption to the wound healing process, this will also facilitate a quicker healing of the wound.

In order to successfully implement wearable sensors for wound monitoring, it is vital to ensure certain characteristics of the sensors, such as high sensitivity, biocompatibility, stability, as well as autonomous functioning and wireless data transmission. The integration of nanomaterials such as carbon-based or metal-based nanoparticles can help increase sensor surface and sensitivity but their use is still controversial, since no relevant information about their long term toxicity and biocompatibility exist for the moment. In the future, research needs to focus on the development of fully autonomous sensors, that ensure wireless data transfer and can function without the need for non-portable devices. This will ensure increased compliance and feasibility of the developed devices. There are currently several limitations regarding the miniaturization of potentiostats, optic probes or batteries that are required for sensor functioning, but consistent effort is being made in order to overcome these challenges. There are already promising examples of autonomous sensors in the literature as well as examples of sensors that can be improved to reach these desired goals.

In order for wearable sensors to be used on a large scale, they need to be intuitive to use and to offer results that are easy to read and interpret. In this context, the integration of smartphones with specially designed applications in sensor use is of great interest. Examples of devices that can yield a result by simply taking a photograph of the sensor or that can offer a naked-eye estimation of different parameters are especially promising for the field of wound infection biomarker monitoring.

Another direction that needs to be taken into consideration in the future is the combination between diagnostic and treatment strategies into the same ‘smart dressing’. Wound dressings that release medicine depending on the concentration of biomarkers present in the wound milieu are of great interest due to their capacity of delivering the substance at exactly the right time.

In conclusion, this review presents the latest advances in the field of wearable and disposable sensors for wound infection biomarker monitoring. The main wound infection biomarkers have been briefly presented and described, followed by a presentation of the most commonly employed methods for biomarker detection, with a focus on electrochemical techniques. Lastly, recent examples of wearable and disposable sensors from the literature have been discussed comparatively.

## Figures and Tables

**Table 1:** Examples of biomarkers for wound infection diagnosis. Adapted from References [13,16].

Wound Infection Biomarkers	
**Physiochemical parameters** pH Oxygenation Temperature Moisture	**Signaling molecules** Nitric oxide Hydrogen peroxide Cytokines Interleukins
**Enzymes** Myeloperoxidase Human-neutrophil elastase Cathepsin G Lysozyme Matrix metaloproteinases Xanthine oxidase α-amylase	**Bacteria** *Pseudomonas aeruginosa* *E. coli* *Bacteroides fragilis* *Staphylococcus aureus* Antibiotic resistant bacteria
**Metabolites** Uric acid Lactic acid	**Bacteria metabolites** Pyocyanin

**Table 2:** Advantages and disadvantages of different types of wearable sensors for wound infection biomarker detection.

Sensor Type	Advantages	Disadvantages
Electrochemical	Low cost fabrication; Low waste fabrication; Possibility of large-scale production; High versatility; High sensitivity	Difficulty in miniaturizing potentiostats; Need for external power sources; Possible electrode fouling in contact with wound fluid
Colorimetric	Low cost fabrication; Intuitive naked-eye estimation; No need for specially designed probes (for smartphone-based approach)	Possible dye biocompatibility issues; Need for specially designed probes (optoelectronic probe approach) Need for special algorithm development (for smartphone-based approach)
Fluorimetric	Intuitive naked-eye estimation	Possible dye biocompatibility issues; Need for bulky UV lamps or fluorescence spectrophotometers
